# Supplementary material for: Intrinsic donor-bound excitons in ultraclean monolayer semiconductors
Source: Nat Commun. 2021 Feb 8;12:871. doi: 10.1038/s41467-021-21158-8 (PMC7870970; doi:10.1038/s41467-021-21158-8)
Supplement: Supplementary file 1 — Supplementary Information [file 41467_2021_21158_MOESM1_ESM.pdf]

Supplementary Information for
Title: Intrinsic Donor Bound Excitons in Ultraclean Monolayer Semiconductors

Authors: Pasqual Rivera[1], Minhao He[1], Bumho Kim[2], Song Liu[2], Carmen Rubio-Verdú[3], Hyowon Moon[4], Lukas Mennel[4], Daniel A. Rhodes[2], Hongyi Yu[5], Takashi Taniguchi[6], Kenji Watanabe[7], Jiaqiang Yan[8,9], David G. Mandrus[8-10], Hanan Dery[11], Abhay Pasupathy[3], Dirk Englund[4], James Hone[2#], Wang Yao[5#], & Xiaodong Xu[1,12#]

Affiliations:

[1]Department of Physics, University of Washington, Seattle, Washington 98195, USA

[2]Department of Mechanical Engineering, Columbia University, New York, NY, 10027, USA

[3]Department of Physics, Columbia University, New York, NY, 10027, USA

[4]Department of Electrical Engineering and Computer Science, Massachusetts Institute of Technology, Cambridge, MA, 02139, USA

[5]Department of Physics, University of Hong Kong, and HKU-UCAS Joint Institute of Theoretical and Computational Physics at Hong Kong, China

[6]International Center for Materials Nanoarchitectonics, National Institute for Materials Science, Tsukuba, Ibaraki 305-0044, Japan.

[7]Research Center for Functional Materials, National Institute for Materials Science, Tsukuba, Ibaraki 305-0044, Japan.

[8]Materials Science and Technology Division, Oak Ridge National Laboratory, Oak Ridge, Tennessee, 37831, USA

[9]Department of Materials Science and Engineering, University of Tennessee, Knoxville, Tennessee, 37996, USA

[10]Department of Physics and Astronomy, University of Tennessee, Knoxville, Tennessee, 37996, USA

[11]Department of Electrical and Computer Engineering, University of Rochester, Rochester, New York, 14627, USA

[12]Department of Materials Science and Engineering, University of Washington, Seattle, Washington, 98195, USA

[#]Correspondence to xuxd@uw.edu; wangyao@hku.hk; jh2228@columbia.edu

Supplementary Figures:
1. **Assignment of free 2D exciton states**
2. **Spatial homogeneity of the satellite emission**
3. **Robust satellite properties across many samples**
4. **Power dependence from additional samples**
5. **Linear polarization resolved satellite emission**
6. **STM/STS measurements of WSe_2 crystals**
7. **Trion excitation dependent donor bound exciton photoluminescence**

Supplementary Notes:
1. **Assignment of donor type defect**
2. **Trion excitation dependent donor bound exciton photoluminescence**

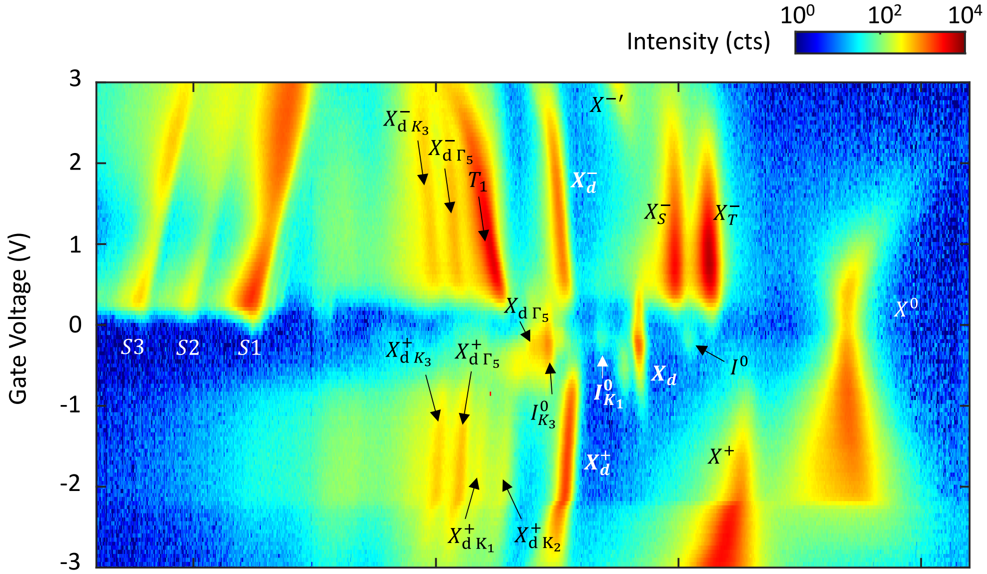

Supplementary Figure 1. Assignment of free 2D excitons in monolayer WSe_2. This is the same data as in Fig. 1c in the maintext, with all reported excitonic feature identified and labeled. At hole doping side (negative gate voltage), from high energy to low energy, the identified excitonic features are: positively charged trion (X^+),[1] positively charged dark trion (X_d^+),[2,3] and K_2, K_1, Γ_5, and K_3 phonon replica of X_d^+ ($X^+_{d\,K2}$, $X^+_{d\,K1}$, $X^+_{d\,\Gamma5}$, $X^+_{d\,K3}$).[4-8] At charge neutral regime, from high energy to low energy, the identified excitonic feature are: bright exciton (X^0),[1] intervalley dark exciton (I^0),[6,7] dark exciton (X_d),[9-14] K_1 and K_3 phonon replica of I^0 ($I^0_{K_1}$, $I^0_{K_3}$),[6] and Γ_5 phonon replica of dark exciton $X_{d\,\Gamma_5}$.[4-8] At electron doping side (positive gate voltage), from high energy to low energy, the identified excitonic features are: negatively charged intervalley (X_T^-) and intravalley trion (X_S^-),[15,16] $X^-{}'$ state,[1] dark trion (X_d^-),[2,3,9-14] T_1 state,[4-14] Γ_5 and K_3 phonon replica of X_d^- ($X^-_{d\,\Gamma5}$, $X^-_{d\,K3}$).[4-8] Note that the nature of the $X^-{}'$ state and the T_1 state are still under active investigation.

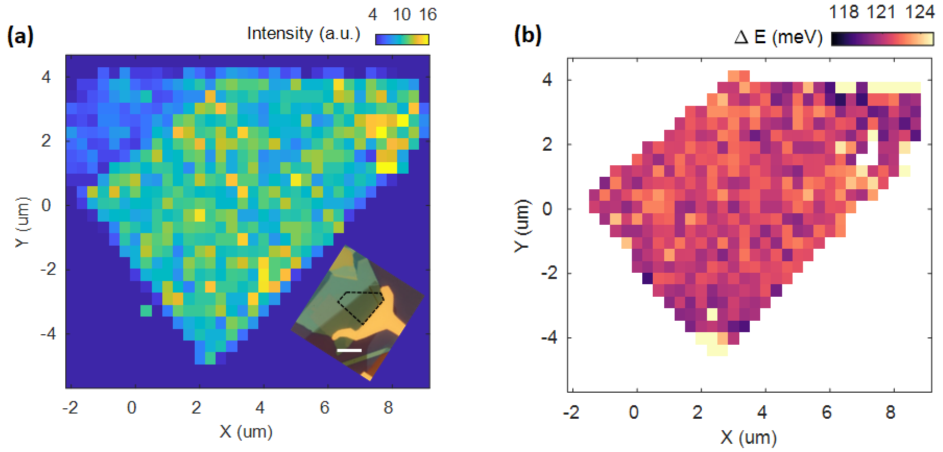

Supplementary Figure 2. Spatial homogeneity of excitonic satellite emission. a, Hyperspectral spatial map of satellite emission intensity, with spatial resolution of 333nm. Inset shows the optical image of the device with same orientation. Monolayer WSe_2 region is marked out by the black dashed line. Scale bar is 10µm. **b**, Hyperspectral spatial map of the energy difference between the first satellite peak (S1) and X^0.

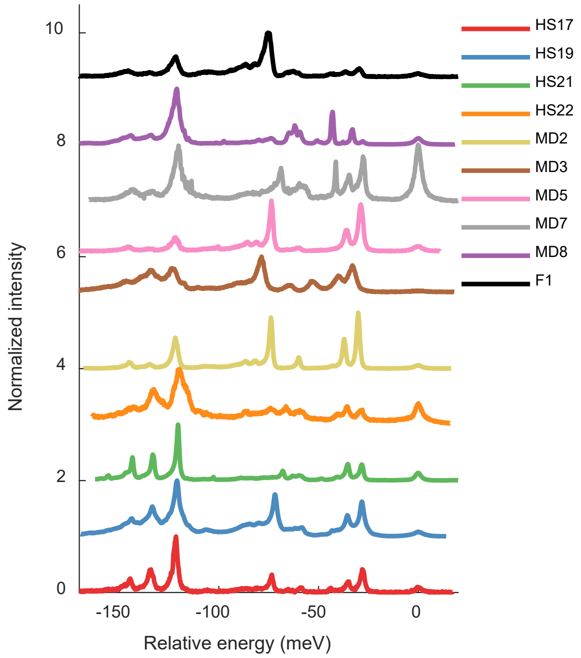

Supplementary Figure 3. Robust satellite properties across 10 samples. Waterfall plot of photoluminescence spectra from 10 different samples, showing homogeneous satellite binding energies and spectra structures. The energy axis is scaled relative to the neutral exciton. Spectrum are taken with no electrostatic gating and all flakes are slightly electron doped due to dilute donors. All samples were fabricated over a 3-year span. Devices HS17, HS19, HS21, HS22, MD2, MD5, MD7 were fabricated from flakes that are exfoliated from multiple crystals grown by vapor transport at ORNL. Devices F1, MD8, and MD3 were fabricated from flakes that are exfoliated from three different batches of crystals by flux growth at Columbia University.

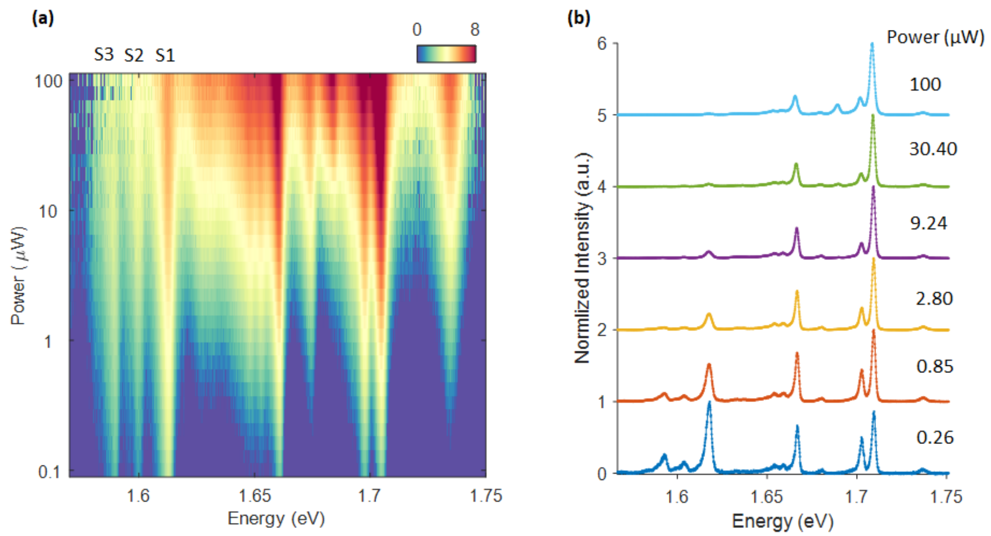

Supplementary Figure 4. Power dependence from additional samples. a, Photoluminescence spectrum as function of excitation power (log scale) in Device 1, at gate voltage of 0.8V. **b,** Waterfall plot of normalized PL spectra at selected excitation powers in Device 1.

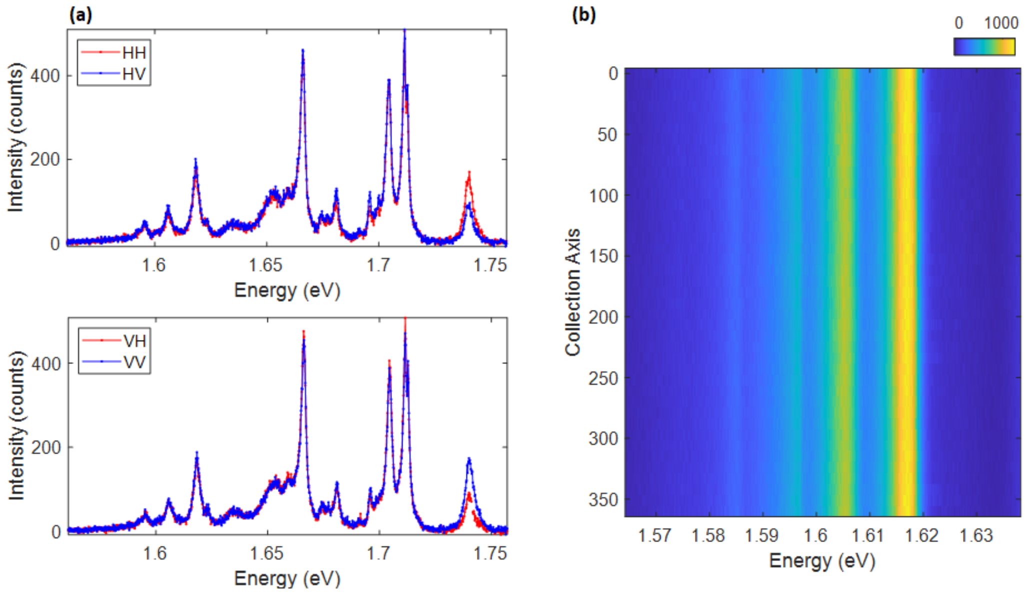

Supplementary Figure 5. Linear polarization resolved satellite emission. a, Photoluminescence spectra with linearly polarized excitation and collection. Data were taken from device HS 17 with 10 μW 633nm CW laser excitation. Top and bottom panels are horizontally (H) and vertically (V) polarized excitation, respectively. **b,** Satellite photoluminescence intensity plot under horizontally polarized excitation with rotating linear polarization collection. Data were taken from device HS19 with 1.5 μW 724nm CW laser

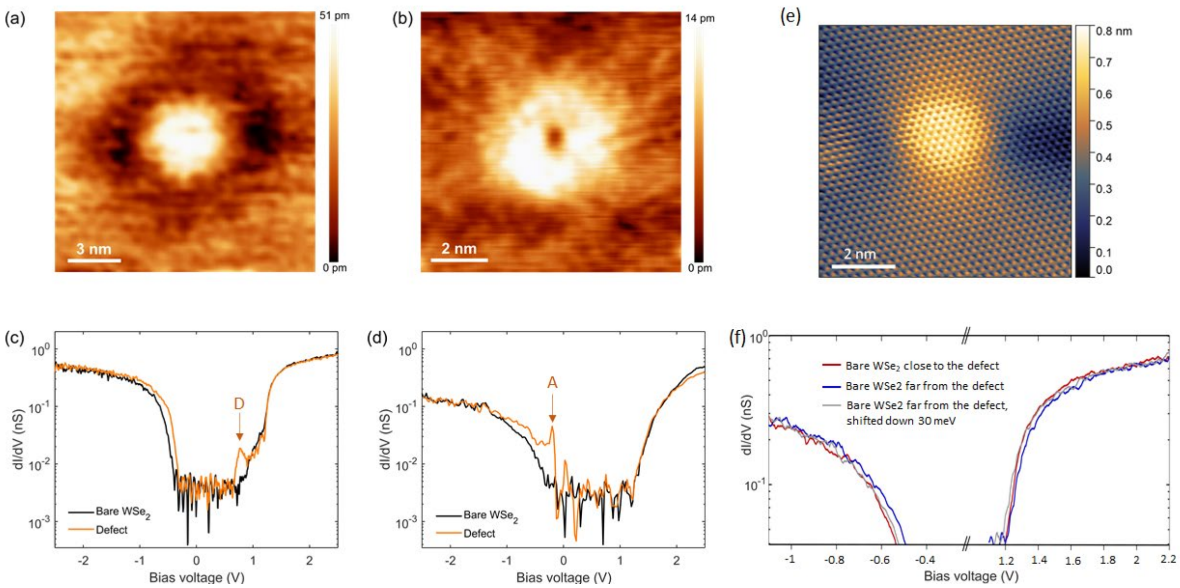

Supplementary Figure 6. Scanning tunneling microscopy and spectroscopy analysis of donor and acceptor defects of WSe_2 bulk crystals at ~10K. STM topographic images of **a**, a donor defect (imaging conditions: 1.4 V, 50 pA) and **b**, an acceptor defect (imaging conditions: 2.0 V, 50 pA). Here, (a) and (b) correspond to the bright and dark defects shown in Fig. 3 in the main text. Differential conductance curves obtained on **c**, a donor defect and **d**, an acceptor defect. Here, D and A denote donor and acceptor bands, respectively. The differential conductance obtained on the bare WSe_2 (black curve) is shown for comparison. The binding energies corresponding to donor and acceptor defects are about 110 meV and 125 meV, respectively. **e**, Atomic resolution STM image of WSe_2 bright donor defect (V=1.4V, I=100 pA) at room temperature. **f**, STS obtained on the WSe_2 surface close (red) and far (blue) from the donor defect. A 30 meV shifted STS is shown in grey line for comparison.

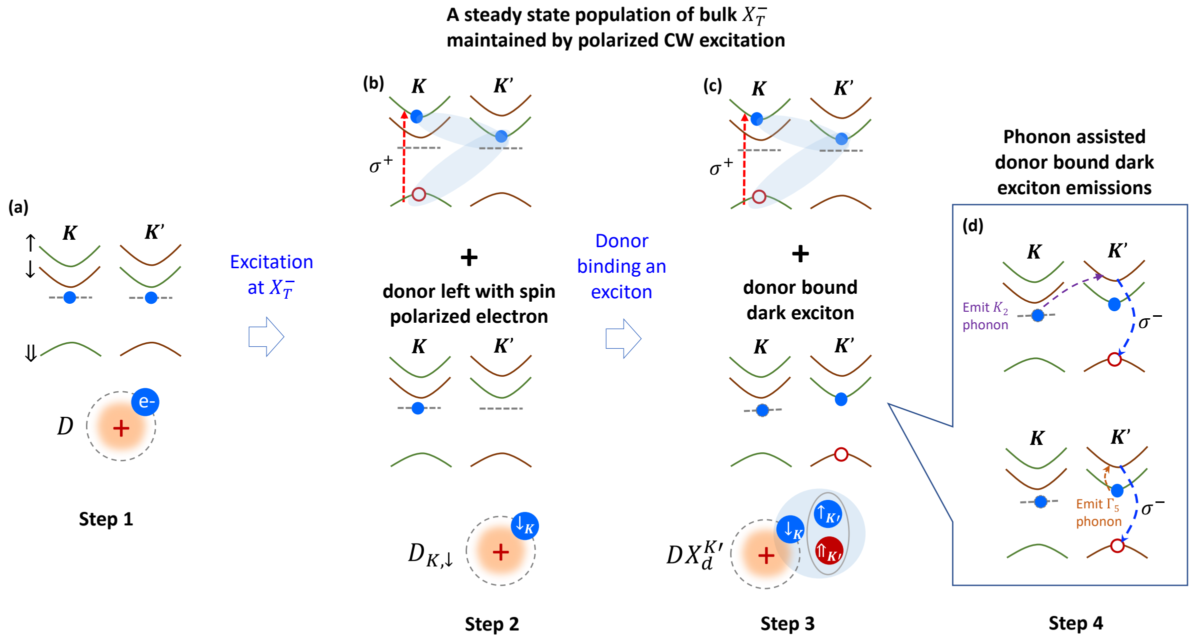

Supplementary Figure 7. Trion excitation dependent donor bound exciton photoluminescence. This is an expanded flow diagram of Fig. 5 in the main text and focuses on intervalley trion excitation with σ^+ polarized light. **a**, step 1 depicts unpolarized donor electrons without optical pumping. **b**, step 2 shows donor spin state initialization by optical pumping of intervalley trion. **c**, step 3 illustrates donor bound exciton formation. **d**, step 4 shows donor bound dark exciton emission assisted by intervalley ($K2$) and intravalley (Γ_5) phonons. See supplementary note 2 for details.

Supplementary Note 1. Assignment of donor type defect.

Here, we present collective experimental evidences that the defect resulted in bound excitons is a donor type. Our gate dependent PL shows that defect bound excitons only appear when the monolayer is electron doped. This implies the defect band of interest is near the conduction band minimum (CBM). As shown by Fig 3 in the maintext, we have examined devices made of different crystals obtained by controllable growths. The devices showing defect bound excitons are slightly n doped, while the device made from crystals without defect bound excitons are slightly p doped. All devices are made by the same fabrication procedure. These facts suggest that the initial n doping is unlikely from trapped impurities between monolayer WSe_2 and hBN introduced during fabrications. STM study shows that only the devices with bright defects (see Figs. 3a &d) produced defect bound excitons. We further performed scanning tunneling spectroscopy (STS) measurements on these defects. Supplementary Figures 6a and 6b show the STM topographic images of the bright and dark defects, as identified in Fig. 3 in the main text. Supplementary Figures 6c and d show the corresponding differential conductance curves. Clearly, for the crystal producing defect bound excitons, we observe a defect band below CBM (Supplementary Fig. 6c), with binding energy of about 110 meV. There are no other bands observed, ruling out the complicated charge levels within the gap. We further obtained STS on the WSe_2 surface close (red) and far (blue) from the donor defect (Supplementary Fig. 6f). Careful analysis of the band edges of the valence and conduction bands on the different locations show that there is an energy offset between them, likely due to band bending. As seen in the figure, the offset between the two curves

is about 30 meV, and corresponds to downward band bending near the defect. This supports the donor nature of the defect with positive charging. The defect binding energy in monolayer will be larger than 110 meV, but should be in the same order of magnitude and thus comparable to exciton binding energy in monolayers.[17-22] This supports the shallow potential of the donor. In addition, the devices host defect bound excitons are slightly n doped at zero gate voltage, evident by the negative gate voltage for the charge neutrality point in gate dependent PL, as illustrated in Fig.1c and Fig. 3d in the maintext. This slightly intrinsic n doping is also consistent with the donor nature of the defect. On the other hand, the devices made of crystal in Fig. 3c, which does not have defect bound excitons, are slightly p doped (see gate dependent PL in Fig. 3f in the maintext). This is consistent with the observed defect band near the valence band maximum in Fig. S6d.

Supplementary Note 2. Trion excitation dependent donor bound exciton photoluminescence. Here we will further clarify Fig. 5 in the maintext. For simplicity, we only focus on the excitation of intervalley trion with σ^+ polarized optical excitation (Supplementary Fig. 7).

Step 1: without optical excitation. Supplementary Figure 7a depicts the valley-spin-coupled band edges and unpolarized donor bound electrons.

Step 2: Donor spin state initialization. As illustrated in the top panel Supplementary Fig. 7b, resonant σ^+ polarized excitation creates $+K$ valley polarized intervalley trion $X_T^-(+K)$. In this continuous wave excitation experiment, maintaining a steady-state population of $X_T^-(+K)$ “uses up” spin up species of the donor electrons. This optical pumping process then leaves the donor with spin down electrons (denoted as $D_{K,\downarrow}$), as shown in the lower panel of Supplementary Fig. 7b.

Step 3: Donor bound exciton formation. The bottom panel of Supplementary Fig. 7c illustrates that the spin polarized donor $D_{K,\downarrow}$ then selectively binds to a dark exciton in the K' valley to form $DX_d^{K'}$, because of the Pauli exclusion between the electrons. Note that a steady state population of the $X_T^-(+K)$ is maintained in this whole process due to σ^+ polarized continuous wave excitation (top panel Supplementary Fig. 7c). Dark exciton is the ground state and its long lifetime makes possible efficient formation of DX_d.

Step 4: Donor bound exciton light emission. $DX_d^{K'}$ can emit light via two process. One is via defect mediated direct electron-hole recombination in the K` valley. The second is via phonon assisted stokes emission (Supplementary Fig. 7d). The top panel of Supplementary Fig. 7d shows intervalley spin-conserved electron scattering assisted by K_2 phonon, which give rises to S2 peak via an intermediate bright trion state. The bottom panel shows the intravalley spin-flip electron scattering assisted by Γ_5 phonon for S3 peak.

Supplementary References

1 A. M. Jones *et al.* "Optical generation of excitonic valley coherence in monolayer WSe_2", *Nat. Nanotech.* **8**, 634(2013).

2 E. Liu *et al.* "Gate Tunable Dark Trions in Monolayer WSe_2", *Phys. Rev. Lett.* **123**, 027401(2019).

3 Z. Li *et al.* "Direct Observation of Gate-Tunable Dark Trions in Monolayer WSe_2", *Nano Letters* **19**, 6886(2019).

4 Z. Li *et al.* "Emerging photoluminescence from the dark-exciton phonon replica in monolayer WSe_2", *Nat. Commun.* **10**, 1(2019).

5 E. Liu *et al.* "Valley-selective chiral phonon replicas of dark excitons and trions in monolayer WSe_2", *Phys. Rev. Res.* **1**, 032007(2019).

6 M. He *et al.* "Valley phonons and exciton complexes in a monolayer semiconductor", *Nat. Commun.* **11**, 618(2020).

7 Z. Li *et al.* "Momentum-Dark Intervalley Exciton in Monolayer Tungsten Diselenide Brightened via Chiral Phonon", *ACS Nano* **13**, 14107(2019).
8 E. Liu *et al.* "Multipath Optical Recombination of Intervalley Dark Excitons and Trions in Monolayer WSe_2", *Phys. Rev. Lett.* **124**, 196802(2020).
9 Y. Zhou *et al.* "Probing dark excitons in atomically thin semiconductors via near-field coupling to surface plasmon polaritons", *Nat. Nanotech.* **12**, 856(2017).
10 M. R. Molas *et al.* "Brightening of dark excitons in monolayers of semiconducting transition metal dichalcogenides", *2D Mater.* **4**, 021003(2017).
11 C. Robert *et al.* "Fine structure and lifetime of dark excitons in transition metal dichalcogenide monolayers", *Phys. Rev. B* **96**, 155423(2017).
12 G. Wang *et al.* "In-plane propagation of light in transition metal dichalcogenide monolayers: optical selection rules", *Phys. Rev. Lett.* **119**, 047401(2017).
13 X.-X. Zhang *et al.* "Magnetic brightening and control of dark excitons in monolayer WSe_2", *Nat. Nanotech.* **12**, 883(2017).
14 M. R. Molas *et al.* "Probing and Manipulating Valley Coherence of Dark Excitons in Monolayer WSe_2", *Phys. Rev. Lett.* **123**, 096803(2019).
15 A. M. Jones *et al.* "Excitonic luminescence upconversion in a two-dimensional semiconductor", *Nat. Phys.* **12**, 323(2016).
16 G. Plechinger *et al.* "Trion fine structure and coupled spin–valley dynamics in monolayer tungsten disulfide", *Nat. Commun.* **7**, 12715(2016).
17 Z. Ye *et al.* "Probing excitonic dark states in single-layer tungsten disulphide", *Nature* **513**, 214(2014).
18 A. Chernikov *et al.* "Exciton Binding Energy and Nonhydrogenic Rydberg Series in Monolayer WS_2", *Phys. Rev. Lett.* **113**, 076802(2014).
19 C. Zhang *et al.* "Direct Imaging of Band Profile in Single Layer MoS_2 on Graphite: Quasiparticle Energy Gap, Metallic Edge States, and Edge Band Bending", *Nano Letters* **14**, 2443(2014).
20 K. He *et al.* "Tightly Bound Excitons in Monolayer WSe_2", *Phys. Rev. Lett.* **113**, 026803(2014).
21 M. M. Ugeda *et al.* "Giant bandgap renormalization and excitonic effects in a monolayer transition metal dichalcogenide semiconductor", *Nat. Mater.* **13**, 1091(2014).
22 G. Wang *et al.* "Giant Enhancement of the Optical Second-Harmonic Emission of WSe_2 Monolayers by Laser Excitation at Exciton Resonances", *Phys. Rev. Lett.* **114**, 097403(2015).
23 Y. J. Zheng *et al.* "Point Defects and Localized Excitons in 2D WSe_2", *ACS Nano* **13**, 6050(2019).
24 S. Barja *et al.* "Identifying substitutional oxygen as a prolific point defect in monolayer transition metal dichalcogenides", *Nat. Commun.* **10**, 3382(2019).
25 C. Palacios-Berraquero *et al.* "Large-scale quantum-emitter arrays in atomically thin semiconductors", *Nat. Commun.* **8**, 15093(2017).
26 A. Branny *et al.* "Deterministic strain-induced arrays of quantum emitters in a two-dimensional semiconductor", *Nat. Commun.* **8**, 15053(2017).
27 Y. Luo *et al.* "Deterministic coupling of site-controlled quantum emitters in monolayer WSe_2 to plasmonic nanocavities", *Nat. Nanotech.* **13**, 1137(2018).
28 M. R. Rosenberger *et al.* "Quantum Calligraphy: Writing Single-Photon Emitters in a Two-Dimensional Materials Platform", *ACS Nano* **13**, 904(2019).
29 S. Kumar *et al.* "Strain-Induced Spatial and Spectral Isolation of Quantum Emitters in Mono- and Bilayer WSe2", *Nano Letters* **15**, 7567(2015).
30 G. Moody *et al.* "Microsecond Valley Lifetime of Defect-Bound Excitons in Monolayer WSe_2", *Phys. Rev. Lett.* **121**, 057403(2018).
31 D. Edelberg *et al.* "Approaching the Intrinsic Limit in Transition Metal Diselenides via Point Defect Control", *Nano Letters* **19**, 4371(2019).
32 D. Han *et al.* "Chemical Trend of Transition-Metal Doping in WSe_2", *Phys. Rev. Appl.* **12**, 034038(2019).
